# Effectiveness of Psychobiotic *Bifidobacterium breve* BB05 in Managing Psychosomatic Diarrhea in College Students by Regulating Gut Microbiota: A Randomized, Double-Blind, Placebo-Controlled Trial

**DOI:** 10.3390/nu16131989

**Published:** 2024-06-22

**Authors:** Yufan Wang, Yufei Wang, Kunpeng Ding, Yuhan Liu, Dingming Liu, Weijun Chen, Xinyi Zhang, Chuanlin Luo, Hongyan Zhang, Tangchang Xu, Tingtao Chen

**Affiliations:** 1 Medical Center of Burn Plastic and Wound Repair, The First Affiliated Hospital, Jiangxi Medical College, Nanchang University, Nanchang 330006, China; 2 Queen Mary School, Jiangxi Medical College, Nanchang University, Nanchang 330031, China; 3 Second College of Clinical Medicine, Nanchang University, Nanchang 330031, China; 4 The Reproductive Hospital, Jiangxi University of Traditional Chinese Medicine, Nanchang 330031, China; 5 School of Life Sciences, Nanchang University, Nanchang 330031, China; 6 National Engineering Research Centre for Bioengineering Drugs and Technologies, Institute of Translational Medicine, Nanchang University, Nanchang 330031, China; 7 School of Pharmacy, Jiangxi Medical College, Nanchang University, Nanchang 330006, China

**Keywords:** *bifidobacterium breve* bb05, dcs, gut microbiota, anxiety and depression, microbiota–gut–brain axis

## Abstract

Diarrhea of college students (DCS) is a prevalent issue among college students, affecting their daily lives and academic performance. This study aims to explore the potential effect of *Bifidobacterium breve* BB05 supplements on the DCS. Initially, fifty healthy and fifty diarrheal students were recruited in the observational experiment and allocated into control and diarrhea groups, respectively. Subsequently, one hundred diarrheal students were newly recruited in the intervention experiment and randomly allocated into placebo and probiotic groups, both treated for 2 weeks. Questionnaires (BSS, HAMA-14, and HDRS-17) were performed to assess the students’ diarrheal states and mental health at baseline and post-treatment. Fecal samples underwent 16S rRNA sequencing and Enzyme-Linked Immunosorbent Assay to evaluate gut microbiota and fecal metabolite alternations. Results indicated that *B. breve* BB05 supplementation significantly enriched (*p* < 0.05) the reduced gut microbial diversity caused by diarrhea. Diarrhea resulted in notable alterations in gut microbiota composition, as exhibited by elevated *Collinsella* and *Streptococcus*, alongside substantially decreased *Bifidobacterium*, *Bacteroides*, and *Prevotella*, while *B. breve* BB05 supplementation partially restored the compromised gut microbiota at both the phylum and genus levels, particularly by increasing *Bifidobacterium* and *Roseburia* (*p* < 0.05). Importantly, questionnaire results suggested that *B. breve* BB05 administration achieved superior efficacy in relieving diarrhea symptoms and the associated anxiety and depression in college students. An increased fecal concentration of 5-hydroxytryptamine (5-HT) was also observed in the probiotic group, while Acetylcholine (ACH), Epinephrine (EPI), and Noradrenaline/Norepinephrine (NANE) reduced, revealing the potential of *B. breve* BB05 in alleviating anxiety and depression via modulating the microbiota–gut–brain axis. Furthermore, correlation analysis suggested that the altered microbiota and fecal neurotransmitters were closely associated with the mental symptoms. These results endorse *B. breve* BB05 intervention as a promising and innovative approach to alleviate both diarrhea and mental health conditions among college students.

## 1. Introduction

The diarrhea of college students (DCS) is characterized by three or more passages of watery or loose stools per 24 h period, which may mainly be attributed to migration, unadaptability to a new environment, and changes in diet [1]. Students suffering from diarrhea may complain of symptoms such as abdominal cramps, vomiting, and fever [2]. Moreover, diarrhea may lower students’ quality of life and academic performance and even give rise to psychological disorders such as anxiety or depression [3,4]. Nowadays, the mainstream therapy for DCS is the replacement of fluid and electrolyte losses using oral rehydration solutions. However, its therapeutic value is limited due to the failure to reduce the diarrheal output and the duration of illness [5]. Therefore, it is urgently required to find an innovative complementary therapeutic regimen with long-term therapeutic effects for DCS.

Previous studies have proposed that gut dysbiosis may be related to diarrhea and mental health disorder including anxiety and depression [6,7]. A clinical study performed by Fujita et al. observed the relative abundance of anaerobic organisms, such as *Bacteroides, Clostridium, Bifidobacterium*, and *Lactobacillus,* was 3–4 times lower in secretory diarrheal illnesses [8]. The changes may be attributed to the disruption of the gut microenvironment caused by the diarrheal state, and commensal bacteria are expelled and replaced by intestinal pathogens [9]. Moreover, recent advances in understanding the microbiota–gut–brain axis have highlighted the role of gut dysbiosis in neurodevelopmental disorders via immune, neuronal, and endocrine pathways [10]. Specially, altered levels of 5-hydroxytryptamine (5-HT), dopamine (DA), and Norepinephrine (NE) in the brain have been observed in rodents with dysbiotic gut microbiota compared with their controls, suggesting an impact of gut dysbiosis on depression levels [11]. The underlying mechanisms may involve the promotion of neurotransmitter synthesis in the central nervous system (CNS) by metabolites such as short-chain fatty acids (SCFAs) produced by gut microbes or direct action through the vagus nerve [12]. Therefore, there is an increased interest in positively regulating the gut microbiota by dietary probiotics to treat diarrhea and associated mental disorders in college students.

Probiotics are defined as live microorganisms that provide a health benefit to the host when administrated in adequate amounts [13]. Consistent evidence demonstrates the therapeutic effect of probiotics on diarrhea through improving the balance of gut microbiota, and the mechanisms may be associated with the competitive exclusion of intestinal pathogens, the production of antimicrobial compounds, and the stimulation of host immune response [14]. Currently, *Bifidobacterium* has shown its role in decreasing the stool frequency and diarrhea duration when compared with placebo [15,16,17]. In addition, *Bifidobacterium* is also confirmed to positively affect the CNS through modulating the microbiota–gut–brain axis [18]. A clinical study demonstrated that probiotic *B. infantis* treatment could reverse depression-like behaviors, which may be associated with the fact that tryptophan secreted by *B. infantis* affects the transmission of brain 5-HT [19]. Collectively, probiotic *Bifidobacterium* may hold a promising future as an adjunct in the treatment of DCS, addressing both somatic and mental aspects.

In this study, we enrolled 50 healthy undergraduates and 150 diarrheal undergraduates to evaluate the efficacy of *Bifidobacterium breve* BB05 on the gastrointestinal symptoms of diarrhea and associated psychological disorders in college students. Questionnaires were used to evaluate the severity of diarrhea, anxiety, and depression among enrolled college students. 16S rDNA high-throughput sequencing and Enzyme-Linked Immunosorbent Assay (ELISA) were used to investigate changes in the gut microbiota and fecal metabolites during diarrhea, respectively. This study aims to fill the knowledge gap and provide new evidence for probiotics as an innovative complementary therapy to the DCS.

## 2. Materials and Methods

### 2.1. Study Design and Ethical Approval

This randomized, double-blind, placebo-controlled trial was conducted from September 2023 to February 2024 in Nanchang University. Volunteer recruitment information was posted online. Following that, students were instructed to contact staff members to be interviewed once they experienced gastrointestinal symptoms of diarrhea. Then, students were asked to complete the Bristol Stool Scale (BSS), the 14-item Hamilton Anxiety Scale (HAMA-14), and the 17-item Hamilton Depression Rating Scale (HDRS-17). Staff members offered comprehensive instructions to students regarding the BSS questionnaire and its application for assessing stool formality following episodes of diarrhea. Upon fecal sample collection, staff members conducted a secondary confirmation of BSS scores with the students. Students with baseline BSS diarrhea scores of 5–7 were identified as having diarrhea, and those with scale scores less than 5 were screened as healthy. The diagnosis, intervention, and potential side effect assessment in this study were all conducted with the involvement of professional gastroenterologists. Participants experiencing exacerbated symptoms or harboring concerns were provided the option to withdraw from the study at any point.

This trial was divided into two stages; the former one was the observational experiment (Stage 1), and the later one was the probiotic intervention experiment (Stage 2). The observational experiment was intended to investigate the severity of diarrhea among college students, diarrhea-associated mental issues, and the alternations in gut microbiota in diarrheal students compared to healthy students. While the subsequent probiotic intervention experiment aimed to evaluate the therapeutic effect of probiotic supplements on diarrheal college students, the restoration of gut microbiota post-treatment, and the alternations of mental-associated fecal metabolites.

The inclusion criteria were as follows: (1) age between 18 and 30 years old; (2) healthy college students or college students who meet the diagnostic criteria for diarrhea; (3) students with diarrhea due to long-distance travel for the new semester, new environment, and alternations in diet; (4) informed consent for this study and voluntary participation.

The exclusion criteria were as follows: (1) previous history of gastrointestinal surgery; (2) patients with a history of irritable bowel syndrome, inflammatory bowel disease, severe allergic disease, constipation, chronic diarrhea, infectious or idiopathic diarrhea, and serious heart, liver, kidney disease, or malignant disease; (3) use of antibiotics, probiotics, prebiotics, laxatives or other drugs known to affect the gut microbiota within the last 3 months; (4) and pregnant or lactating patients.

All the participants in this study signed the informed consent form. All procedures were approved and complied with Institutional Ethics Committee on medical research of the Nanchang Reproductive Hospital (Examination and approval No. 2023096). This study was registered in the Chinese Clinical Trial Registry (chictr.org.cn) with the registration number ChiCTR2300074826. The Declaration of Helsinki, the World Health Organization’s (WHO) worldwide ethical principles, and the China Good Clinical Practice principles (Issue 57, 2020) were all closely followed during this trial.

### 2.2. Randomization and Masking

In the observational experiment (Stage 1), healthy participants were assigned to the healthy control group (C group), and diarrheal participants were assigned to the diarrhea group (M group). In the intervention experiment (Stage 2), participants with diarrhea were assigned in a 1:1 ratio to the probiotic group (MB group) and the placebo group (MP group) using the random number table method. A non-participating trial staff member then allocated random numbers to the probiotics and placebos. Then, dedicated medication management staff administered either the probiotic agent or placebo to participants based on their assigned random number. There were no significant differences in packaging, color, or odor between probiotics and placebo, thus ensuring a double-blind status between the investigator and patient. It is worth noting that the product provider neither provided financial support for the trial nor played any role in the trial’s conceptualization or execution.

### 2.3. Intervention Procedure and Management

Observational experiment (Stage 1): Initially, students were recruited, and those with a BSS score below 5 were identified as healthy individuals. Concurrently, to screen for diarrheal students, participants were instructed to promptly inform staff members upon experiencing diarrhea symptoms. Staff members would then promptly assess them, and those with a BSS score between 5 and 7 were categorized as diarrheal students. Out of those 100 students, 50 healthy students were assigned as the C group, and another 50 students with diarrhea were regarded as the M group. The subjects in each group were of the same age, and the ratio of male to female was 1:1. The validated questionnaires were provided to determine their diarrhea and mental conditions at baseline. The participants were guided to collect fecal samples independently at home using a stool sampling tube according to the manufacture’s instructions. The fecal sample was promptly obtained by a staff member and preserved at −80 °C for high-throughput sequencing.

Intervention experiment (Stage 2): Out of 100 newly recruited students with diarrhea, 50 were selected randomly as the MB group to receive the probiotic product, and the other 50 receiving the placebo product were regarded as the MP group. The participants in the MB and MP group were instructed to ingest the probiotic and placebo product twice daily for 2 consecutive weeks, respectively. The experimental probiotic was *B. breve* BB05 (China General Microbiological Culture Collection Center No.: 24982) with 1 × 10^10^ colony-forming units per packet of live bacteria. The placebo contained only the same grams of maltodextrin. *B. breve* BB05 (product number: 20230809) and the placebo (product number: 20230802) were provided by Thankcome Biological Science and Technology (SuZhou) Co., Ltd. (Suzhou, China), and were stored in a refrigerator at 4 °C. The questionnaires were provided to evaluate their diarrhea and mental conditions at Week 0, Week 1, and Week 2. Stool samples were collected according to the above instructions at Week 2 for subsequent high-throughput sequencing and ELISA.

### 2.4. Questionnaires

The appearance of diarrhea was evaluated by using the defecation questionnaires (stool form and frequency). Fecal frequency was assessed by recording defecation times each day. Fecal characteristics were assessed at each defecation using the Bristol Stool Scale (BSS), as follows: Value of 1 (separate hard lumps, like nuts), 2 (“sausage-shaped” but lumpy), 3 (like a sausage but with cracks on its surface), 4 (like a sausage or snake, smooth and soft), 5 (soft blobs with clear-cut edges), 6 (fluffy pieces with rugged edges, a mushy stool), and 7 (watery, no solid pieces) [20]. Further defecation questionnaires included days of defecation in the past one week, times of defecation in the past one week, days of abdominal pain, and the extent of abdominal pain.

The severity of the anxiety symptoms was measured using the Chinese version of the 14-item of Hamilton Anxiety Scale (HAMA-14). The cut-offs for the HAMA-14 standard scores were defined as follows: Normal (0–7), mild or probable anxiety (8–14), moderate or definite anxiety (15–21), and severe anxiety (≥22). The depressive symptoms of students were assessed using the Chinese version of the 17-item Hamilton Depression Rating Scale (HDRS-17). The severity of the depression was classified according to the following severity range of the HAMD-17 score: Normal (0–7), mild depression (8–16), moderate depression (17–23), and severe depression (>23) [21].

### 2.5. Enzyme-Linked Immunosorbent Assay (ELISA)

The concentration of 4 fecal metabolites associated with diarrhea was analyzed by using ELISA after the placebo or probiotic intervention. Based on the method of Cooke et al. [22], 100 mg of stool sample stored at −80 °C was placed in a 1.5 mL EP tube. Then, 0.9 mL of PBS buffer (pH 7.4, 0.01 mol/L) was added. The mixture was thoroughly shaken and centrifuged at 4 °C at 3000 rpm for 15 min. The supernatant was collected and aliquoted. An appropriate amount of the supernatant was taken as needed for ELISA determination, and the remaining samples were stored at −80 °C for future use. The supernatant was analyzed for 5-hydroxytryptamine (5-HT ELISA Kit, J&L, Shanghai, China), Acetylcholine (ACH ELISA Kit, Elabscience, Wuhan, China), Epinephrine (Epinephrine/Adrenaline ELISA Kit, Elabscience, Wuhan, China), and Noradrenaline (Noradrenaline/Norepinephrine ELISA Kit, Elabscience, Wuhan, China) according to the manufacturer’s instructions. Antibodies, standards, dilutions, washing solutions, chromogenic substrates, and a 96-well detection plate were provided in the kit. Hundred μL of sample or standard were added into each well in the plate leaving at least one well for background or blank evaluation. Hundred μL of enzyme-labeled antibody working solution was then added into the sample control and standard wells. The plate was then sealed with a sealing paper and incubated at 37 °C for 60 min in a constant temperature chamber. The absorbance was measured using a CMax Plus absorbance microplate reader (Meigu Molecular Instruments (Shanghai) Co., Ltd., Shanghai, China) to quantify sample concentrations.

### 2.6. DNA Extraction and 16S rRNA Sequencing

Total genomic DNA from the collected stool samples (observational experiment and intervention experiment) was extracted using a Bacterial Genomic DNA Extraction Kit (Solarbio Science & Technology Co., Ltd., Beijing, China) following the manufacturer’s instructions. The concentration and purities of the extracted genomic DNA were determined using a spectrophotometer (NanoDrop; Thermo Fisher Scientific, Inc., Waltham, MA, USA). Extracted DNA was subjected to 16S sequencing at Shanghai Personal Biotechnology Co., Ltd. (Shanghai, China). The V4 regions of the 16S rDNA were amplified for each sample using primers of 515F/806R (515F,5′-GTGCCAGCMGCCGCGGTAA-3′; 806R,5′-TACNVGGGTATCTAATCC-3′), and these PCR products were sequenced with an Illumina HiSeq 2000 platform (Illumina, California, USA). FLASH was applied to merge overlapped reads, and sequence analysis was performed using UPARSE software package (V7.1). Reads with quality scores lower than 20, ambiguous bases, and improper primers were discarded prior to clustering. Simultaneously, chimaeras were checked and eliminated during clustering. The resulting high-quality sequences were clustered into operational taxonomic units (OTUs) at 97% similarity. According to the annotation of taxa, the relative abundance of total bacteria in each sample was categorized into different classification levels (Phylum, Class, Order, Family, and Genera). The alpha diversity index (Shannon index, Observed_species index, and Chao1 index) was calculated and analyzed using QIIME2 software (2019.4) and subjected to intergroup comparisons using the Wilcoxon test. The beta diversity index (principal component analysis, PCoA) was assessed by Bray–Curtis distance. Taxonomic composition analysis at phylum and genus level was mapped with QIIME2 software (2019.4). The abundance of individual species at the phylum or genus level between the two groups was compared using the non-parametric Mann–Whitney U-test.

### 2.7. Sample Size

In the observational experiment, the data from a pre-trial conducted by our research group to assess diarrhea severity among students revealed a BSS score of 4.66 ± 0.63 for healthy students and 5.67 ± 0.75 for those with diarrhea. Using a two-sample *t*-test with an α value of 0.05 (two-tailed) and 95% power, the sample size was estimated at 14 participants per group. Considering factors such as sample sizes normally applied in studies investigating diarrhea [15,23], and its impact on the efficacy of high-throughput sequencing, an initial sample size of 100 participants, with 50 in each group, was determined.

In another pre-trial conducted prior to the intervention experiment, participants were treated with either placebo or *B. breve* BB05 for 2 weeks. The 2-week BSS scores for the placebo and probiotic groups were 4.40 ± 0.69 and 3.82 ± 0.71, respectively. Utilizing a two-sample *t*-test with an α value of 0.05 (two-tailed) and 95% power, the sample size was estimated at 39 participants per group. Assuming a potential attrition rate, dropout rates, and other unforeseen contingencies of 20% among participants, an initial sample size of 100 was ultimately determined for the intervention experiment, with 50 individuals allocated to each group.

### 2.8. Statistical Analysis

Continuous variables were presented as mean ± standard deviation, with normality assessed using the Shapiro–Wilk test and variance equality assessed using Levene’s test. To evaluate the questionnaire scores between healthy and diarrheal students in the observational experiment, we employed the Mann–Whitney U-test. The Kruskal–Wallis test was performed to assess the questionnaire scores before and after treatment in the intervention experiment. Significant alterations in fecal neurotransmitters were determined through the Mann–Whitney U-test. Spearman correlation analysis was conducted to assess the relationship between diarrhea, anxiety, and depression scores and the key altered microbial species, as well as the correlation among the modified fecal neurotransmitters. Statistical analysis and data visualization were performed using Prism software version 9.5.1 (GraphPad Software, San Diego, CA, USA), QIIME2 software (2019.4), R 4.2.3 software, PASS 15.0, and SPSS 26. And the significance level was set at *p* < 0.05.

## 3. Results

### 3.1. Diarrhea Affects Mental Health and Gut Microbiota on College Students (Observational Experiment)

#### 3.1.1. Baseline Characteristics and Scales Results of Participants in the Observational Experiment

**Figure 1:**
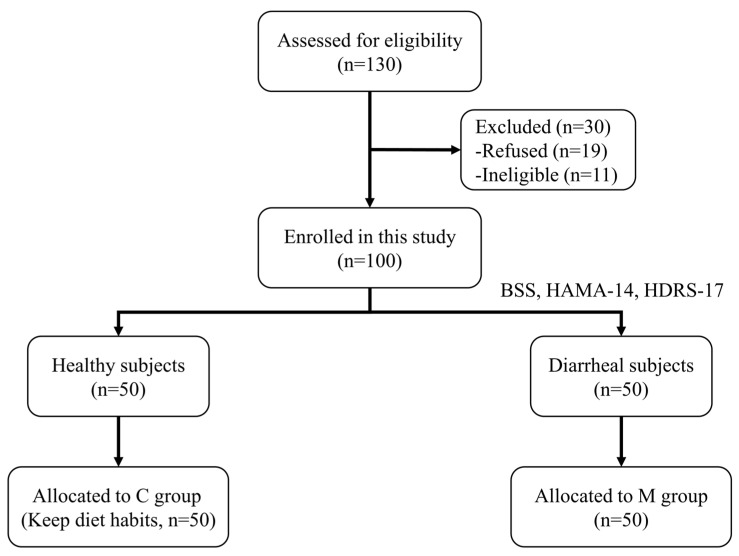
**Study flowchart of the observational experiment (Stage 1).** BSS: Bristol Stool Scale; HAMA-14: 14-item of Hamilton Anxiety Scale; HDRS-17: 17-item Hamilton Depression Rating Scale. C: The healthy control group; M: The diarrheal group.

A total of 130 students underwent initial enrollment for eligibility assessment in the observational experiment (Stage 1), as illustrated in Figure 1. Based on questionnaire results, 50 students identified with diarrhea were assigned to the M group, and 50 healthy students constituted the C group. The remaining 30 students were excluded from the observational experiment, with 19 refusing participation and 11 meeting the exclusion criteria.

**Figure 2:**
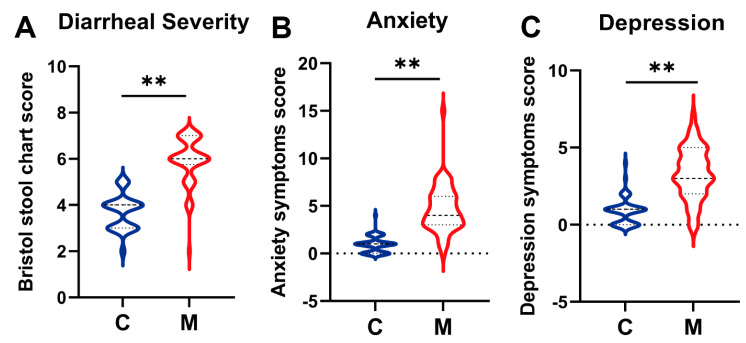
**Questionnaire scores in the observational experiment in terms of BSS, HAMA-14, and HDRS-17.** (**A**) Bristol stool chart score based on BSS. (**B**) Anxiety symptoms score based on HAMA-14. (**C**) Depression symptoms score based on HDRS-17. C: The healthy control group (*n* = 50); M: The diarrheal group (*n* = 50). Questionaries scores were analyzed using the Mann–Whitney U-test. **: *p* < 0.01.

Table 1 outlines the baseline characteristics and questionnaire scores of participants in the observational experiment. No significant differences in age, Body Mass Index (BMI), and gender were observed among the students in the C group and M groups (*p* > 0.05, Table 1). The BSS, HAMA-14, and HDRS-17 questionnaires were administered to assess students’ diarrheal and mental health conditions at baseline. The BSS fecal characteristic scores were 3.70 and 5.90 in the C group and M groups, respectively, aligning with the criteria for healthy and diarrheal conditions (Table 1, Figure 2A). In terms of mental health, the HAMA-14 anxiety and HDRS-17 depression scale scores were 4.60 and 3.33 in the M group, while they were 1.00 and 0.86 in the C group (Table 1, Figure 2B,C). These suggest that the occurrence of diarrhea may elicit mild anxiety and depression among college students.

#### 3.1.2. Diarrhea and Perturbance in Gut Microbial Diversity and Composition in College Students

Further, to investigate the association between diarrhea and the gut microbiota of college students, stool samples were collected from both healthy controls (C group) and students experiencing diarrhea (M group) for 16S rRNA-seq analysis.

**Figure 3:**
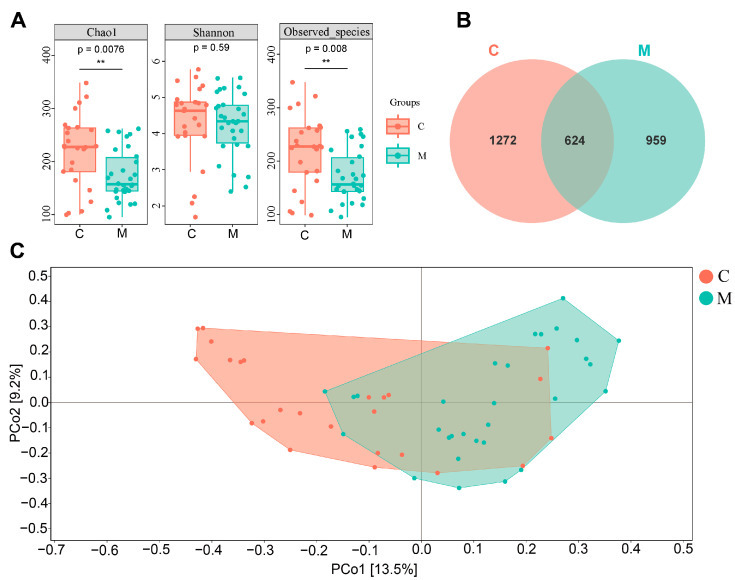
**Diarrhea and reduced gut microbial diversity.** (**A**) Chao1, Shannon, and Observed_species indices of the gut microbiota. (**B**) Venn diagram of gut microbial species. (**C**) PCoA analysis of gut microbiota. C: The healthy control group (*n* = 25); M: The diarrheal group (*n* = 30). The alpha diversity indexes were analysed using the Wilcoxon test, while PCoA analysis was assessed by bray_curtis distance. **: *p* < 0.01.

In the α-diversity analysis (Figure 3A), a notable difference in gut microbial richness was observed in the M group compared to the C group, as indicated by significantly decreased Chao 1 (*p* = 0.0076) and Observed_species (*p* = 0.008) indices. Although the Shannon index (*p* = 0.59) demonstrated no significant difference, a consistent trend indicated decreased species diversity in the gut of the M group relative to the C group. These α-diversity disparities (Figure 3A) demonstrated the alterations in microbial diversity, signifying that diarrhea may be associated with the altered equilibrium of the normal human gut microbiota. Furthermore, the Venn diagram (Figure 3B) demonstrated 624 shared operational taxonomic units (OTUs) across the C and M groups, with unique 959 OTUs in the M group and 1272 in the C group. This indicates a correlation between the occurrence of diarrhea and alternations in the types of gut microbiota species. Additionally, PCoA analysis (Figure 3C) indicated that the microbial samples between the C group and the M group were manifestly discrete, further revealing the close interconnection between diarrhea and the structural composition of the human gut microbiota.

**Figure 4:**
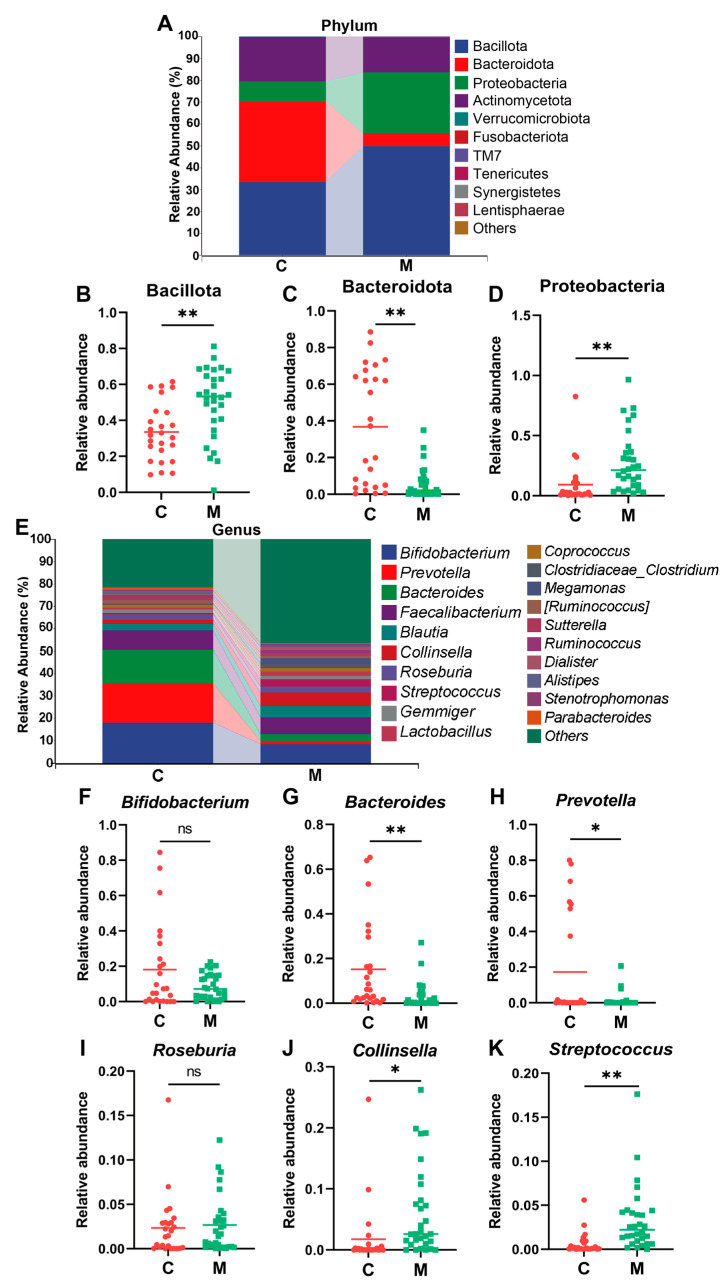
**Diarrhea and perturbance in gut microbial composition.** (**A**) The relative abundance of gut bacteria at the phyla level. (**B**–**D**) The relative abundance of Bacillota, *Bacteroidota*, and Proteobacteria. (**E**) The relative abundance of gut bacteria at the genus level. (**F**–**K**) The relative abundance of *Bifidobacterium*, *Bacteroides*, *Prevotella*, *Roseburia*, *Collinsella*, and *Streptococcus*. C: The healthy control group (*n* = 25); M: The diarrheal group (*n* = 30). The abundance of individual species at the phylum or genus level between two groups was compared using the Mann–Whitney U-test. *: *p* < 0.05; **: *p* < 0.01; ns: not significant.

At the phylum level (Figure 4A), the predominant microbial populations were Bacillota (Firmicutes), Bacteroidota (Bacteroidetes), Proteobacteria, and Actinomycetota (Actinobacteria), constituting 99.72% and 99.58% of the total sequencing results in the C and M groups, respectively. Specifically, the relative abundance of Bacillota (Figure 4B) was significant higher in the M group (59.87%, *p* = 0.0013) compared to the C group (33.57%), while Bacteroidota (Figure 4C) was significant lower in the M group (5.72%) relative to the C group (36.81%, *p* < 0.001), indicating the vulnerability of Bacteroidota to diarrhea. Furthermore, Proteobacteria (Figure 4D) exhibited a substantial rise in the M group (28.03%) compared to the C group (9.20%, *p* < 0.01).

At the genus level (Figure 4E), selected representative gut microbiota species underwent detailed analysis. Notably, the relative abundance of *Bifidobacterium* (Figure 4F) was substantially lower in the C group relative to the M group (C vs. M = 18.12% vs. 8.59%, *p* = 0.668), indicating a diminution of beneficial gut microbiota following diarrhea. Additionally, *Bacteroids* (Figure 4G) (C vs. M = 15.13% vs. 3.23%, *p* = 0.003) and *Prevotella* (Figure 4H) (C vs. M = 17.32% vs. 1.36%, *p* = 0.029) also exhibited significant reductions in the C group compared to the M group. The relative abundance of *Roseburia* (Figure 4I) was slightly higher in the M group (2.67%) relative to the C group (2.34%, *p* = 0.366). Remarkably, the opportunistic pathogens *Collinsella* (Figure 4J) (C vs. M = 1.76% vs. 6.07%, *p* = 0.002) and *Streptococcus* (Figure 4K) (C vs. M = 0.63% vs. 3.28%, *p* < 0.01) were significantly higher in the M group compared to the C group. Collectively, the above results suggest that diarrhea may be correlated with an increase in pathogenic microbial populations and a reduction in beneficial gut microbiota, implying a compromised gut microbial barrier and a more vulnerable microenvironment.

### 3.2. B. breve BB05 Intervention Improves Gut Dysbiosis and Mental Health in Diarrheal College Students (Intervention Experiment)

#### 3.2.1. Baseline Characteristics and The impact of *B. breve* BB05 on Diarrhea Symptoms and Associated Anxiety and Depression

**Figure 5:**
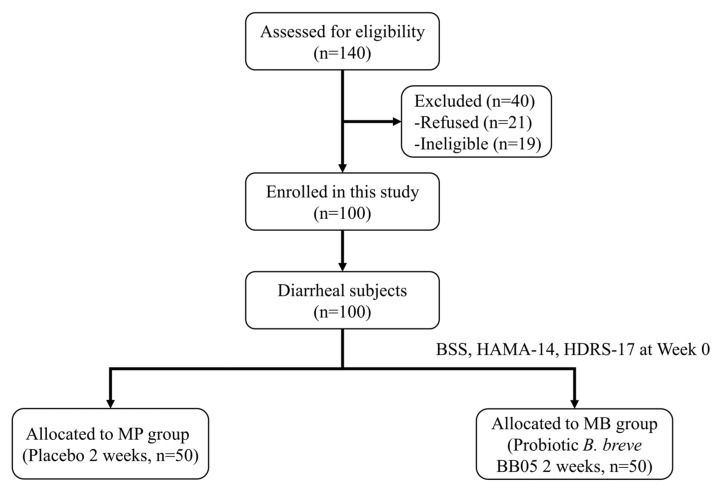
**Study flowchart of the probiotic intervention experiment (Stage 2).** BSS: Bristol Stool Scale; HAMA-14: 14-item of Hamilton Anxiety Scale; HDRS-17: 17-item Hamilton Depression Rating Scale. MP: The MP group taking placebo; MB: The MB group taking *B. breve* BB05.

In order to evaluate the efficacy of *B. breve* BB05 supplements on diarrheal students, a total of 140 students were newly enrolled in the intervention experiment (Stage 2), as illustrated in Figure 5. Following questionnaire results, 100 students screened with diarrhea were evenly assigned (50:50) to the MP group and the MB group for the intervention experiment. The remaining 40 students were excluded from the intervention experiment (21 declined to participate, and 19 met the exclusion criteria).

**Figure 6:**
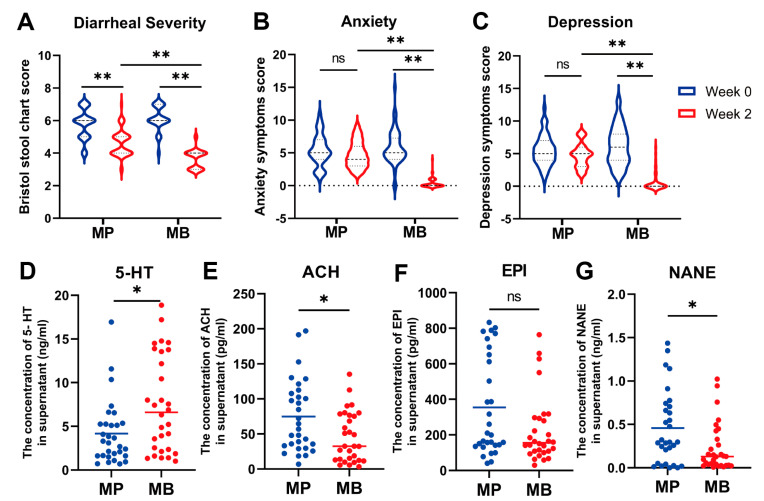
Questionnaire scores in the intervention experiment and the effects of *Bifidobacterium breve* BB05 on fecal neurotransmitters. (**A**) Bristol stool chart score based on BSS. (**B**) Anxiety symptoms score based on HAMA-14. (**C**) Depression symptoms score based on HDRS-17. (**D**) The concentration of 5-HT. (**E**) The concentration of ACH. (**F**) The concentration of EPI. (**G**) The concentration of NANE. 5-HT: 5-hydroxytryptamine; ACH: Acetylcholine; EPI: Epinephrine; NANE: Noradrenaline/Norepinephrine. MP: The MP group taking placebo; MB: The MB group taking *B. breve* BB05. Sample size of questionaries (*n*): MP:MB = 50:50; ELISA (*n*): MP:MB = 30:30. Questionnaire scores were analyzed using the Kruskal–Wallis test, while results from ELISA were analyzed using the Mann–Whitney U-test. *: *p* < 0.05; **: *p*< 0.01; ns: not significant.

Table 2 outlines the baseline characteristics and questionnaire scores of participants in the intervention experiment. At baseline, there was no significant difference in background features in terms of age, BMI, and gender among the students in the MP group and MB groups (*p* > 0.05, Table 2). In line with the observational experiment, students with diarrhea in the intervention experiment exhibited mild anxiety and depression at week 0, suggesting a positive correlation between diarrhea and levels of anxiety and depression. Although both the MP and MB groups showed a significant decrease in BSS fecal characteristic scores after 2 weeks of placebo or probiotic treatment, the MP group exhibited a significantly higher score compared to the MB group at week 2 (MP vs. MB = 4.62 vs. 3.66, *p* < 0.01) (Table 2, Figure 6A), indicating a superior efficacy of the *B. breve* BB05 supplement in diarrhea treatment. Moreover, in the MB group, the HAMA-14 anxiety and HDRS-17 depression scale scores significantly decreased from 5.86 ± 2.65 and 6.12 ± 2.98 at week 0 to 0.38 ± 0.75 and 0.58 ± 1.37 at week 2 (Table 2, Figure 6B,C), whereas the MP group also showed a decrease but without a statistically significant change. These results collectively suggest the potential of the probiotic *B. breve* BB05 supplement in improving diarrhea symptoms and ameliorating physiological conditions such as anxiety and depression.

To further investigate the impact of probiotic supplements on anxiety and depression, the concentration of related fecal neurotransmitters (ACH, 5-HT, EPI, and NANE) was determined by ELISA on the fecal supernatant. In the MB group, the concentration of 5-HT significantly increased (MP vs. MB = 4.18 ng/mL vs. 7.51 ng/mL, *p* = 0.016) compared to the MP group, while the concentration of ACH significantly decreased (MP vs. MB = 75.00 pg/mL vs. 45.69 pg/mL, *p* = 0.014) in the MB group relative to the MP group (Figure 6D,E). These findings indicate an improvement in anxiety and depression from the perspectives of neurotransmitters among college students. Additionally, comparing the MB group to the MP group (Figure 6F,G), the concentration of EPI (MP vs. MB = 0.228 pg/mL vs. 224.2 pg/mL, *p* = 0.074) and NANE (MP vs. MB = 0.456 ng/mL vs. 45.69 ng/mL, *p* = 0.043) was also considerably lower, suggesting probiotic treatment could alleviate students’ anxiety. In summary, the *B. breve* BB05 supplement demonstrated the ability to increase the fecal concentrations of 5-HT while reducing ACH, EPI, and NANE, potentially relieving the diarrhea-induced anxiety and depression.

#### 3.2.2. *B. breve* BB05 Supplement Enriches and Improves the Compromised Gut Microbiota in Diarrheal Students

Furthermore, to assess the alternations of the *B. breve* BB05 supplement on diarrheal gut microbiota, a total of 60 fecal samples were randomly collected for 16S rRNA-seq analysis (30 from the MP group and 30 from the MB group).

**Figure 7:**
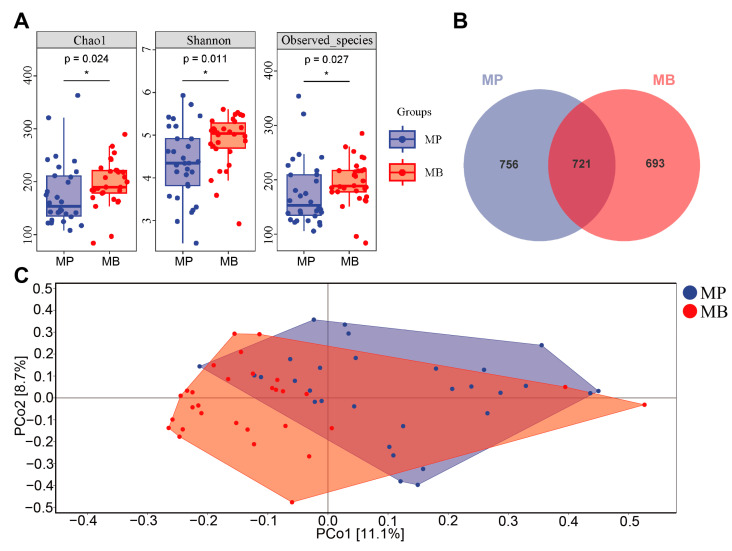
***Bifidobacterium breve* BB05 can enrich gut microbial diversity after diarrhea.** (**A**) Chao1, Shannon, and Observed_species indices of the gut microbiota. (**B**) Venn diagram of gut microbial species. (**C**) PCoA analysis gut microbiota. MP: The MP group taking placebo (*n* = 30); MB: The MB group taking *B. breve* BB05 (*n* = 30). The alpha diversity indexes were analyzed using the Wilcoxon test, while PCoA analysis was assessed by Bray–Curtis distance. *: *p* < 0.05.

In the α-diversity analysis (Figure 7A), Chao 1 (*p* = 0.024), Shannon (*p* = 0.011), and Observed_species (*p* = 0.027) indexes were all significantly increased in the MB group compared to the MP group. These results suggest that the *B. breve* BB05 supplement profoundly enriched the compromised gut microbial diversity following diarrhea. The Venn diagram (Figure 7B) illustrated 721 common OTUs between the two groups, with unique OTU numbers of 756 and 693 for the MP and the MB group, indicating that *B. breve* BB05 intervention could reshape gut microbiota post-diarrhea. Further, the β-diversity analysis through PCoA (Figure 7C) revealed that most samples in the MB group were prominently distant from those in the MP group, suggesting that *B. breve* BB05 could effectively remodel the perturbed gut microbiota.

**Figure 8:**
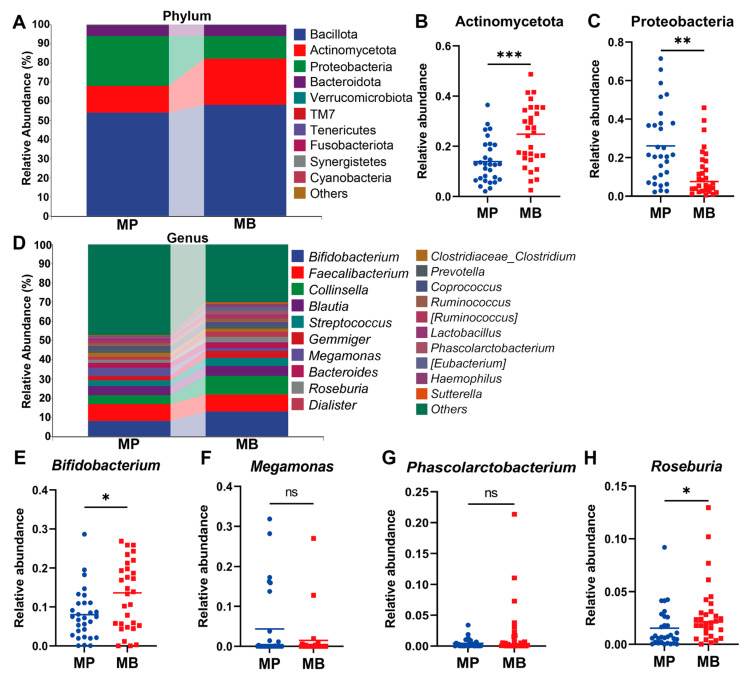
***Bifidobacterium breve* BB05 can improve gut microbial composition after diarrhea.** (**A**) The relative abundance of gut bacteria at the phyla level. (**B**,**C**) The relative abundance of Actinomycetota and Proteobacteria. (**D**) The relative abundance of gut bacteria at the genus level. (**E**–**H**) The relative abundance of *Bifidobacterium*, *Megamonas*, *Phascolarctobacterium*, and *Roseburia*. MP: The MP group taking placebo (*n* = 30); MB: The MB group taking *B. breve* BB05 (*n* = 30). The abundance of individual species at the phylum or genus level between two groups was compared using the Mann–Whitney U-test. *: *p* < 0.05; **: *p* < 0.01; ***: *p* < 0.001; ns: not significant.

At the phylum level (Figure 8A), Bacillota, Actinomycetota, Proteobacteria, and Bacteroidota were the predominant taxa in the students’ gut, accounting for 99.52% and 99.68% of the sequencing results in the MP group and the MB group, respectively. Following *B. breve* BB05 treatment, the MB group exhibited a significantly higher relative abundance of Actinomycetota (MP vs. MB = 13.91% vs. 24.03%, *p* = 0.0007) compared to the MP group (Figure 8B), indicating a reversal of the decline in Actinomycetota under diarrheal conditions observed in the observational experiment. Moreover, Proteobacteria, which was up-regulated under diarrhea conditions, was significantly down-regulated (MP vs. MB = 26.07% vs. 11.88%, *p* = 0.0012) in the MB group than the MP group (Figure 8C), suggesting a partial restored gut microenvironment could be achieved with *B. breve* BB05 intervention. The relative abundance of Bacteroidota was not influenced by probiotic treatment, while the relative abundance of Bacillota was slightly higher in the MB group relative to the MP group.

At the genus level (Figure 8D), the relative abundance of *Bifidobacterium* (Figure 8E), which had substantially decreased under diarrheal conditions, showed a notable enrichment (MP vs. MB = 8.02% vs. 12.92%, *p* = 0.04) following *B. breve* BB05 administration. Additionally, *Roseburia* (MP vs. MB = 1.53% vs. 2.88%, *p* = 0.017) and *Phascolarctobacterium* (MP vs. MB = 0.44% vs. 1.89%, *p* = 0.11) were also markedly more abundant in the MB group post-probiotic supplement than in the MP group (Figure 8G,H), signifying an increase in beneficial gut microbiota after the *B. breve* BB05 supplement. Furthermore, compared to the MP group, the MB group exhibited a reduced relative abundance of *Megamonas* (MP vs. MB = 4.33% vs. 1.46%) (Figure 8F). These results suggest that *B. breve* BB05 has the potential to counteract the alternations by diarrhea in gut microbiota and retrieve the gut homeostasis. Taken together, these findings indicate that a 2-week *B. breve* BB05 treatment could contribute to improving the gut dysbiosis caused by diarrhea, thereby reinstating the gastrointestinal microbiota diversity, particularly by increasing *Bifidobacterium*, *Roseburia*, and *Phascolarctobacterium* and decreasing *Megamonas*.

### 3.3. Correlation Analysis among Phenotypes, Gut Microbiota, and Related Fecal Neurotransmitters

**Figure 9:**
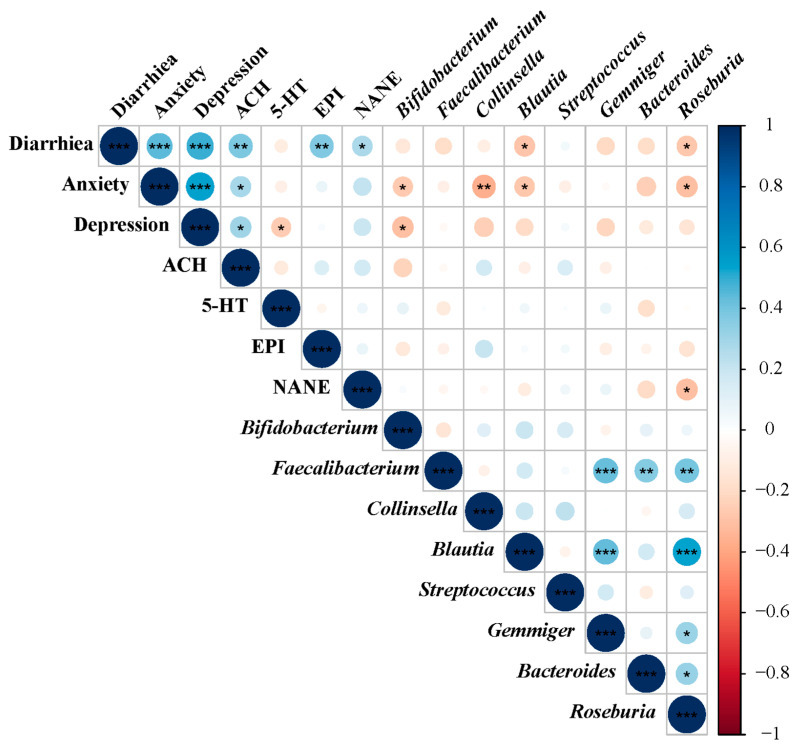
**Spearman analysis among phenotypes, related fecal metabolites, and gut microbiota.** Spearman’s rank correlation coefficient among 3 phenotypes (diarrhea, anxiety, and depression), 4 fecal metabolites, and top 8 relative abundance of gut microbiota. 5-HT: 5-hydroxytryptamine; ACH: Acetylcholine; EPI: Epinephrine; NANE: Noradrenaline/Norepinephrine. *p* values are shown as red and blue, where a negative correlation is represented by red and a positive correlation by blue. *: *p* < 0.05; **: *p* < 0.01; ***: *p* < 0.001.

The Spearman correlation analysis (Figure 9) was then conducted to delve into the intricate relationship between the gut microbiota (top 8 genus), four fecal neurotransmitters (ACH, 5-HT, EPI, and NANE), and diarrhea, anxiety, and depression symptoms. The findings revealed a positive association between diarrhea and the occurrence of anxiety and depression among college students. Additionally, diarrhea displayed a negative correlation with beneficial gut microbiota, including *Bifidobacterium*, *Roseburia*, *Blautia*, *Faecalibacterium*, and *Bacteroides*, while exhibiting a positive correlation with *Streptococcus*. This suggests that a diarrheal condition may detrimentally impact beneficial gut microbiota and potentially promote the proliferation of certain opportunistic pathogens. Furthermore, *Faecalibacterium* demonstrated significant positive correlations with *Roseburia*, *Bacteroides*, and *Gemmiger*, which, in turn, exhibited a significant positive correlation with *Blautia*. Notably, the anxiety condition was negatively correlated with *Bifidobacterium*, *Collinsella*, *Blautia*, and *Roseburia*, with *Bifidobacterium* also showing significant negative correlation with depression. Moreover, the genera *Bifidobacterium* displayed negative associations with ACH and EPI, while being negatively associated with 5-HT. *Roseburia*, on the other hand, exhibited a remarkable association with EPI and NANE. Furthermore, ACH, EPI, and NANE showed positive correlations with both anxiety and depression, whereas 5-HT displayed negative correlations with anxiety and depression. This intricate interplay among microbiota, neurotransmitters, and mental symptoms may imply a potential connection between gut microbiota and psychiatric symptoms through the microbiota–gut–brain axis. In summary, these results suggest strong links between the alterations in gut microbiota induced by diarrhea and the changes in fecal neurotransmitters, potentially contributing to the manifestation of anxiety and depression symptoms among college students.

## 4. Discussion

Diarrhea is prevalent among college students, often attributed to the stress of adapting to a new school environment or abrupt changes in dietary habits, significantly impacting both their physical and mental well-being [24,25,26]. Notably, probiotics have been proposed as a promising strategy in the treatment of diarrhea. In this study, our findings reveal that college students experiencing diarrhea exhibit psychological disorders and gut microbiota dysbiosis compared to the C group. Importantly, a subsequent two-week supplementation of *B. breve* BB05 demonstrates a reduction in diarrhea severity by restoring the diversity and composition of gut microbiota. Additionally, our results indicate that probiotic intervention has a positive impact on alleviating anxiety and depression induced by diarrhea, suggesting the beneficial psychological effects of probiotics through modulating the microbiota–gut–brain axis [27,28].

Previous studies have established a close association between gastrointestinal diseases (e.g., IBS) and psychological disorders [29]. In our study, health questionnaires, including BSS, HAMA-14, and HDRS-17, were employed to assess anxiety and depression levels in college students with diarrhea [30,31,32]. Results showed that students with higher BSS scores also demonstrated mild anxiety and depression (Table 1, Figure 2), suggesting the extent of diarrhea might be positively associated with the level of anxiety and depression. Moreover, our observational experiment demonstrated a significant reduction in α-diversity and deviation of β-diversity in the M group compared to the C group (Figure 3), which indicated a serious loss of bacterial species post-diarrhea. Subsequent investigation revealed a significantly decreased relative abundance of *Bifidobacterium*, *Bacteroides*, and *Prevotella*, a slight reduction in *Roseburia*, and a significant increment of *Collinsella* and *Streptococcus* in diarrheal college students compared to healthy individuals (Figure 4). These findings suggest the potential of restoring gut microbiota balance as a promising strategy for addressing the DCS and associated mental health issues.

Probiotic intervention has shown its role in reestablishing gut microbiota and further reducing the incidence of diarrhea [33]. In light of the findings in the observational experiment that the relative abundance of *Bifidobacterium* was substantially decreased, *B. breve* BB05 was selected for the intervention experiment. We found that the supplement of *B. breve* BB05 significantly improved the diarrhea condition and alleviated anxiety and depression into normal levels. This result was consistent with the effects of *B. breve* in other investigations. Choi et al. showed outcomes in treating diarrhea by modulating diverse pro-inflammatory factors [34]. Tian et al. also found that *B. breve* reduced the severity of major depression disorders by modulating the gut microbiome and tryptophan metabolism [35]. Notably, even though students in the placebo group experienced remission of diarrhea after two weeks, those in the probiotic group achieved lower scores on BSS, suggesting a closer approximation to normal stool characteristics (Table 2, Figure 6). Furthermore, after the 2-week *B. breve* BB05 supplement, both α-diversity and β-diversity were substantially improved in the probiotic group relative to the placebo group (Figure 7), supporting the notion that an enriched gut microbiota diversity may be associated with a healthy physiological state. This is in line with the findings of Lai et al. [23], who demonstrated that probiotic administration could restore the disturbed gut microbiota composition in children with diarrhea, specifically by increasing *Bifidobacterium* at the genus level. Furthermore, the observational experiment revealed a noteworthy lower abundance of Bacteroidota and a notable higher abundance of Proteobacteria at the phylum level following diarrhea (Figure 4). In contrast, post-probiotic supplementation depicted an inverse pattern (Figure 8), indicating a potential partial restoration of dysregulated gut microbiota by *B. breve* BB05 supplementation. Our data further demonstrated increased relative levels of *Phascolarctobacterium* and *Roseburia*, along with a reduction in the abundance of *Megamonas* following *B. breve* BB05 supplementation (Figure 8). *Phascolarctobacterium* and *Roseburia* are known to positively influence gut health and human mood [36]. On the other hand, an increase in *Megamonas* has been widely reported in many diseases such as IBS, obesity, colon cancer, and psychiatric disorders including depression [37]. Altogether, these results indicate that *B. breve* BB05 may improve diarrhea and related psychiatric conditions through enhancing the balance of gut microbiota.

Attention has lately been drawn to the microbiota–gut–brain axis, which refers to a bidirectional relationship between the gut and brain [38]. Gut microorganisms can affect the CNS through modulating neuroendocrine and immune channels, while the CNS can directly influence the gut microbiome via stress-mediator-induced virulence gene expression or through the automatic nervous system [39]. Recent investigations have implicated three primary pathways in the interconnections of the microbiota–gut–brain axis: the immune pathway, the endocrine pathway, and the neuronal pathway. Gut microbiota can interact with the immune system through various mechanisms, including its residence within the gut and the production of microbial-derived metabolites such as SCFAs and amino acid metabolites [38]. SCFAs, among the most extensively studied microbial metabolites, play a crucial role in regulating tight junctions [40] and mitigating damage to the gut epithelium [41]. Consequently, they reinforce the intestinal immunological barriers, thereby safeguarding against pathogenic invasion. Conversely, the disruption of the intestinal barrier by gut dysbiosis permits luminal microbes to access dendritic cell extensions. Immune agonists released by bacteria, such as lipopolysaccharide and peptidoglycans, can then activate Toll-like receptors and initiate immune responses, which have been associated with the pathogenesis of age-related neurodegenerative diseases like Alzheimer’s disease and mood disorders such as depression and anxiety [12]. In addition to modulating the intestinal barrier, the gut microbiota is also implicated in the translocation of immune cells between the gut and the brain. Sanmarco et al. [42] reported that the gut microbiota could facilitate the migration of IFNγ^+^ NK cells into the CNS and induce the proliferation of a type of anti-neuroinflammatory astrocytes. Furthermore, meningeal IgA^+^ plasma cells, crucial for preventing brain infection, were found to be diminished in the CNS of germ-free (GF) mice and later confirmed to originate from the gut [43].

Moreover, the gut microbiota influences the function of the microbiota–gut–brain axis by producing various numerous neurotransmitters and neuromodulators, such as gamma-aminobutyric acid (GABA), 5-HT, ACH, and histamine [44]. Microbial metabolites like 5-aminovaleric acid (5AV) [45] and tetrahydrobiopterin (BH4) [46] have also been associated with sociability in the brain. Apart from endogenously produced metabolites, gut microbes interact with the enteroendocrine system to regulate neurodevelopment. For instance, they can synthesize SCFAs, including butyrate, propionate, and acetate, which boost the synthesis of 5-HT by intestinal enterochromaffin cells through the stimulation of tryptophan hydroxylase 1. Peripheral 5-HT can then indirectly influence the CNS by interacting with 5-HT3 and 5-HT4 receptors located on vagal afferent fibers, thereby facilitating neurodevelopment [47]. Furthermore, the gut microbiota may also contribute to regulating neurodevelopment through its involvement in various microglial events. Bruckner and colleagues proposed that a healthy gut microbiota could modulate the microglial expression of the complement signaling pathway and the synaptic remodeling factor complement C1q [48]. This regulation promotes synaptic pruning and contributes to the development of positive social behaviors.

Therefore, given the significance of gut microbes on gut–brain axis, the effect of probiotics on maintaining intestinal microbial homeostasis may suggest their potentials to regulate brain health via the microbiota–gut–brain axis. Guida et al. reported that gut dysbiosis led to an inflammatory state, altered neuronal hippocampal firing, and depression-like behaviors, with reversal of these phenotypes following probiotic treatment [49]. Similarly, Sushma and colleagues suggested that *B. breve* administration could alleviate depression-like behaviors [50]. Underlying mechanisms may include the improvement of immune system and gut barrier, as well as the production of neurologically active substances such as GABA and SCFAs. Our study also demonstrated that *B. breve* BB05 can restore disturbed gut microbiota in college students with depression and anxiety, supporting its potential in treating mental disorders through modulation of the microbiota–gut–brain axis.

This study further delved into the gastrointestinal concentrations of four fecal neurotransmitters (5-HT, ACH, EPI, and NANE) to explore the underlying mechanisms of probiotic supplementation in alleviating anxiety and depression (Figure 6). 5-HT, a neurotransmitter vastly secreted by enterochromaffin cells (ECs) in the gastrointestinal tract, plays a crucial role in anxiety and depression, with dysfunctions strongly correlated with these conditions [51]. Tian et al. [35] suggested *B. breve* application can alleviate depression along with correlated gastrointestinal symptoms by decreasing the turnover of serum 5-HT, which is consistent with our results. The underlying mechanism by which *B. breve* BB05 affects 5-HT secretion might be due to the modulation of metabolites, such as short-chain fatty acids and secondary bile acids, which can activate ECs to secrete 5-HT [52]. Moreover, the concentrations of ACH, EPI, and NANE were considerably reduced following probiotic supplementation in our study. ACH is a neurotransmitter that is crucial in memory-sustaining and has also been reported to increase under depression or anxiety conditions through facilitating the encoding and maintenance of stressful memories [53]. And the increase in EPI and NANE induced by the over-activation of the sympathetic nervous system during physical or emotional trauma can also contribute to anxiety behaviors [54]. According to our results from correlation analysis (Figure 9), the occurrence of diarrhea was negatively correlated with diverse beneficial microbiota, including *Bifidobacterium* and *Roseburia*, which were also negatively correlated with the concentration of ACH, NANE, and EPI. These results imply that diarrhea may elicit the reduction in specific bacteria, contributing to the dysregulation of neurotransmitters and, consequently, anxiety and depression. Conversely, *B. breve* BB05 supplementation appears could restore gut microbiota balance, significantly associated with the reduction in anxiety and depression. Collectively, our results support the hypothesis that *B. breve* BB05 supplementation improves psychiatric disorders through the microbiota–gut–brain axis by regulating neurotransmitter concentrations.

Although the effect of *B. breve* BB05 in restoring damaged gut microbiota has been demonstrated in this study, uncertainties remain regarding the contributions of individual differences or probiotic administration to its efficacy. Since this trial was a dietary intervention involving university students, the evaluation of diarrhea was limited to the BSS diarrhea score. This may present some limitations to the results of our study. While the observational experiment provided valuable insights, the absence of fecal samples collected before probiotic or placebo intervention hinders this study to directly compare microbiota recovery before and after probiotic intervention. Consequently, this study can only suggest that *B. breve* BB05 supplementation may improve compromised gut microbiota post-diarrhea, rather than definitively asserting that probiotics can restore it. Another major limitation is that, while students were confirmed not to have used probiotic products 3 months before the experiment and were informed not to use them during the experiment, it is challenging to monitor the actual use of probiotic products in practice. This could lead to potential variations in the gut microbiota induced by diet. In the observational study, the relative abundance of *Bifidobacterium* was noticeably lower after diarrhea, although not statistically significant. This could potentially be attributed to the small sample size, and a larger sample size might better highlight this difference. Moreover, assessing the therapeutic efficacy of *B. breve* BB05 on DCS and mental health disorders would benefit from a more extended follow-up period. Additionally, the particularity of the college population makes it difficult to collect serum samples, so fecal samples were collected instead. Although a previous study [55] has utilized fecal neurotransmitter concentrations, it remains unclear whether these levels accurately reflect systemic concentrations. This uncertainty further complicates the explanation of the relationship between fecal mental metabolites and diarrhea. Further, SCFAs in feces are an important factor that should not be overlooked. Existing studies have demonstrated their association with the composition of the gut microbiota. An increased abundance of SCFA-producing bacteria can promote sodium and water absorption in the colonic epithelium, effectively ameliorating diarrhea by modulating nutrient absorption and intestinal function [56,57]. However, the role of SCFAs in DCS remains unclear. Further exploration of the relationship between SCFAs and DCS in subsequent clinical studies is crucial. This will help propose more precise and effective strategies for the treatment of DCS. Finally, the underlying mechanism of *B. breve* BB05 in alleviating anxiety and depression could be investigated by stress-induced animal models and cutting-edge molecular techniques, specifically in its partnership with the microbiota–gut–brain axis. This aspect may emerge as a crucial focus for the application of probiotics in future clinical management.

## 5. Conclusions

**Figure 10:**
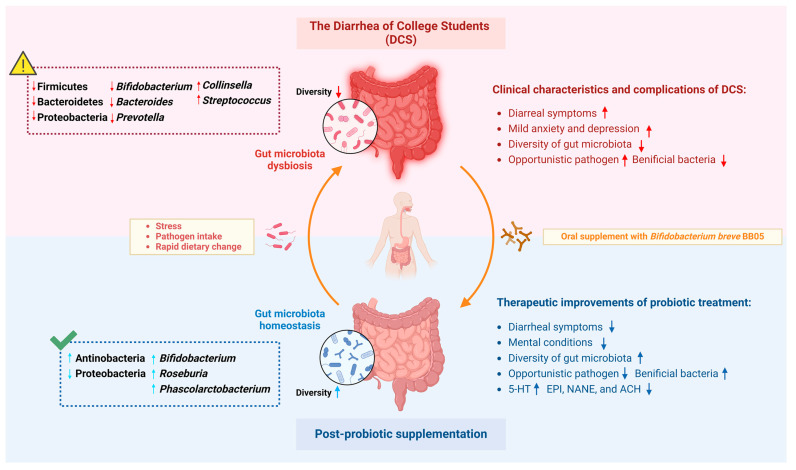
Schematic diagram of the effect of *Bifidobacterium breve* BB05 on clinical symptoms of the diarrhea on college students and associated gut microbiota dysbiosis. 5-HT: 5-hydroxytryptamine; ACH: Acetylcholine; EPI: Epinephrine; NANE: Noradrenaline/Norepinephrine. Figure 10 was created using Biorender (biorender.com)

In conclusion, the present study observed the significant impact of DCS on the structure and composition of the gut microbiota in college students, potentially contributing to their anxiety and depression symptoms (Figure 10). The subsequent intervention experiment reveals the effectiveness of probiotic supplementation with *B. breve* BB05 in treating diarrheal symptoms and alleviating associated psychological disorders among college students. Specifically, probiotic administration could restore the compromised gut microbiota under diarrhea-induced stress and remodulate the mental-correlated fecal neurotransmitters (Figure 10). Moreover, a robust correlation emerges between diarrhea-induced mental symptoms and the altered gut microbiota species and fecal neurotransmitters. This suggests the potential utility of these biomarkers in the diagnosis and treatment of various mental health diseases. Our findings offer novel insights for clinicians regarding the application of probiotics in treating diarrhea and promoting gut health among college students. Still, long-term follow-up and larger-scale studies are needed to comprehensively understand the role of *B. breve* BB05 in the microbiota–gut–brain axis and unveil its therapeutic potential in managing both diarrhea and psychiatric disorders.

## Figures and Tables

**Table 1:** Baseline characteristics and scales results of participants in the observational experiment (Stage 1).

Characteristics	C Group (*n* = 50)	M Group (*n* = 50)	*p* Value
Age	19.70 ± 0.92	19.4 ± 0.86	/
BMI	22.34 ± 1.97	22.58 ± 1.19	/
Female:Male (*n*:*n*)	1:1 (25.00:25.00)	1:1 (25.00:25.00)	/
HAMA-14 ^a^	1.00 ± 0.91	4.60 ± 3.03 **	<0.01
HDRS-17 ^b^	0.86 ± 0.93	3.33 ± 1.88 **	<0.01
BSS ^c^	3.70 ± 0.76	5.90 ± 1.03 **	<0.01

Data are shown as mean ± SD; *n* refers to number; BMI, Body Mass Index. All *p*-values calculated using the Mann–Whitney test. ^a^ The 14-item Hamilton Anxiety Scale (HAMA-14) was used to assess the anxiety symptoms. ^b^ The 17-item Hamilton Depression Rating Scale (HDRS-17) was used to evaluate the depressive symptoms. ^c^ The Bristol Stool Scale (BSS) was used to assess the severity of diarrhea.**: *p* < 0.01.

**Table 2:** Baseline characteristics and scales results of participants in the intervention experiment (Stage 2).

Characteristics	MP Group (*n* = 50)		*p* Value	MB Group (*n* = 50)		*p* Value
	Week 0	Week 2		Week 0	Week 2	
Age	19.43 ± 1.04		/	19.63 ± 0.81		/
BMI	22.40 ± 1.72		/	21.77 ± 1.64		/
Female:Male (*n*:*n*)	1:1 (25.00:25.00)		/	1:1 (25.00:25.00)		/
HAMA-14 ^a^	5.42 ± 2.41	4.74 ± 1.99 ^ns^	0.1314	5.86 ± 2.65	0.38 ± 0.75 **	<0.01
HDRS-17 ^b^	5.50 ± 2.32	4.70 ± 1.81 ^ns^	0.0865	6.12 ± 2.98	0.58 ± 1.37 **	<0.01
BSS ^c^	5.78 ± 0.91	4.62 ± 0.83 ***	<0.001	6.10 ± 0.76	3.66 ± 0.59 **	<0.01

Data are shown as mean ± SD; *n* refers to number; BMI, Body Mass Index. All *p*-values calculated using the Mann–Whitney test. ^a^ The 14-item Hamilton Anxiety Scale (HAMA-14) was used to assess the anxiety symptoms. ^b^ The 17-item Hamilton Depression Rating Scale (HDRS-17) was used to evaluate the depressive symptoms. ^c^ The Bristol Stool Scale (BSS) was used to assess the severity of diarrhea. **: *p* < 0.01; ***: *p* < 0.001. ns refers to no significance.

## Data Availability

The raw reads were preserved in the Sequence Read Archive (SRA) database of NCBI. The accession numbers can be found below: PRJNA1084101 (SRA).
